# Nicotine facilitates VSMC dysfunction through a miR-200b/RhoGDIA/cytoskeleton module

**DOI:** 10.1038/srep43798

**Published:** 2017-03-02

**Authors:** Dongli Liang, Zhaoxia Wang, Zhiqiang Yan, Shangwei Hou, Wangjie Xu, Lianyun Wang, Meisheng Shang, Zhongdong Qiao

**Affiliations:** 1 School of Life Sciences and Biotechnology, Shanghai Jiao Tong University, Shanghai, 200240, PR China; 2 Laboratory Animal Center of Instrumental Analysis Center, Shanghai Jiao Tong University, Shanghai, 200240, PR China; 3 Central laboratory, Shanghai Jiao Tong University affiliated Sixth People’s Hospital South Campus, Shanghai, 201400, PR China; 4 Hongqiao international institute of medicine, Tongren Hospital, Shanghai Jiao Tong University School of Medicine, Shanghai, 200336, PR China; 5 Beijing Anzhen Hospital, Capital Medical University, Beijing, 100029, PR China

## Abstract

Nicotine can induce the abnormal migration and proliferation of vascular smooth muscle cells (VSMCs). We have previously shown that cytoskeletal proteins and RhoGDIA, a negative regulator of the Rho GTPase pathway, are involved in the nicotine-induced dysfunction of VSMCs. Here, we found that nicotine can activate the Rho GTPase pathway and induce the synthesis of the cytoskeletal proteins in VSMCs through the activation of intracellular downstream signaling pathways, including targets such as MYPT1, PAK1 and PI3K/AKT. Upon nicotine treatment, the mRNA level of RhoGDIA is increased but protein level is decreased both *in vitro* and *in vivo*, which suggested a mechanism of post-translational regulation. By the dual luciferase reporter assay, we identified the microRNA-200b (miR-200b) as a modulator of the behavioural changes of VSMCs in response to nicotine through targeting RhoGDIA directly. Introducing miR-200b inhibitors into cultured VSMCs significantly attenuated cell proliferation and migration. Additionally, we found that hypomethylation in the CpG island shore region of *miR-200b* was responsible for the nicotine-induced miR-200b up-regulation in VSMCs. The study demonstrates that nicotine facilitates VSMC dysfunction through a miR-200b/RhoGDIA/cytoskeleton module through the hypomethylation of *miR-200b* promoter and suggests that epigenetic modifications may play an important role in the pathological progression.

Cigarette smoke is a complex mixture of more than 4000 chemicals and cigarette smoking is considered to be a major risk factor for the development of cardiovascular diseases^1^,^2^ such as atherosclerosis and hypertension. It is reported that nicotine, an addictive agent in cigarette smoke, has a wide variety of effects on vascular dysfunction^3^. For example, nicotine can induce the migration and proliferation of vascular smooth muscle cells (VSMCs)^4^,^5^,^6^,^7^,^8^. However, the molecular mechanisms underlying this pathological process have not been elucidated.

Abnormalities in the structure of the actin cytoskeleton are associated with various cellular processes^9^ and are also involved in the nicotine-induced migration and proliferation of VSMCs^6^,^10^. Our previous study demonstrated that nicotine could affect the expression of four cytoskeleton proteins in VSMCs, including β-actin, γ-actin, tropomyosin 4 and F-actin, and as well as RhoGDIA (Rho-specific guanine nucleotide dissociation inhibitor A)^11^. RhoGDIs inhibit the GTPase activity of Rho proteins by suppressing the release of the nucleotide and preventing both intrinsic and GAP-stimulated hydrolysis of GTP. So far, three evolutionarily conserved mammalian RhoGDIs have been identified, RhoGDIA (α), RhoGDIB (β) and RhoGDIC (γ)^12^. Among them, RhoGDIA is the most abundant and well-characterized member of the family. RhoGDIA can interact with several Rho GTPases, including RhoA, RAC1, RAC2 and CDC42^12^. The pathological changes induced by nicotine in VSMCs are very likely to be associated with the Rho GTPase family, which plays a central role in many diverse biological processes such as actin cytoskeleton organization, microtubule dynamics, gene transcription and cell cycle progression^13^. However, the molecular mechanisms by which RhoGDIA or Rho GTPases contribute to nicotine-induced dysfunction of VSMCs have not been elucidated.

MicroRNAs (miRNAs) are small, endogenous non-coding RNAs that can post-transcriptionally regulate gene expression^14^. miRNAs bind to the 3′-untranslated regions (3′-UTRs) of target mRNAs to either prevent their translation or cause degradation of the target^15^. Deregulation of miRNAs is involved in a wide range of human diseases, including cardiovascular diseases and cancers^16^,^17^. Recent studies have shown that various functions in VSMCs are finely regulated by miRNAs^18^, including miR-143/miR-145^19^,^20^, miR-21^21^, miR-31^22^ and miR-221/miR-222^23^,^24^. However, it has not been reported whether miRNAs are involved in the nicotine-induced dysfunction of VSMCs. In addition, although miRNAs play a critical role in regulating RhoGDIA in several diseases^25^, the mechanism by which RhoGDIA is regulated by miRNAs in VSMCs after nicotine exposure is not well defined.

Epigenetic modifications, such as DNA methylation and histone modifications, are key determinants of chromatin structure and gene expression^26^,^27^. These modifications are maintained during cell division and when perturbed, play a key role in cell development^28^. Among them, hypermethylation or hypomethylation of the promoter region is a common mechanism underlying aberrant gene expression at the transcriptional level^29^. Recent studies have shown that nicotine can induce changes in DNA methylation in several diseases^30^,^31^,^32^. Nevertheless, it has not been clarified whether nicotine can cause gene deregulation by DNA methylation in VSMCs.

Therefore, in the current study, we investigated whether RhoGDIA is involved in promoting migration and proliferation of VSMCs in response to nicotine exposure and we sought to determine the mechanism underlying nicotine-induced deregulation of RhoGDIA.

## Results

### The Rho GTPase pathway is involved in the change of cytoskeletal protein expression induced by nicotine

In accordance with previous reports^33^, VSMCs were treated with different concentrations (10^−9^, 10^−8^, 10^−7^, 10^−6^, 10^−5^ and 10^−4^ M) of nicotine for various amounts of time (0, 1, 12, 24 and 48 h). As shown in Supplementary Figs S1 and S2, nicotine at a concentration of 10 μM for 24 h or longer could most heavily decrease the expression of RhoGDIA, so VSMCs were collected 24 h after treatment with 10 μM nicotine in the study. Meanwhile, to determine whether the Rho GTPase pathway was associated with nicotine-induced deregulation of proteins, α-BtX (α7-nicotinic acetylcholine receptor, α7-nAchR specific antagonist) and Y27632 (an inhibitor of Rho-Kinase activation) were added to the culture media 30 min before nicotine treatment.

**Figure 1:**
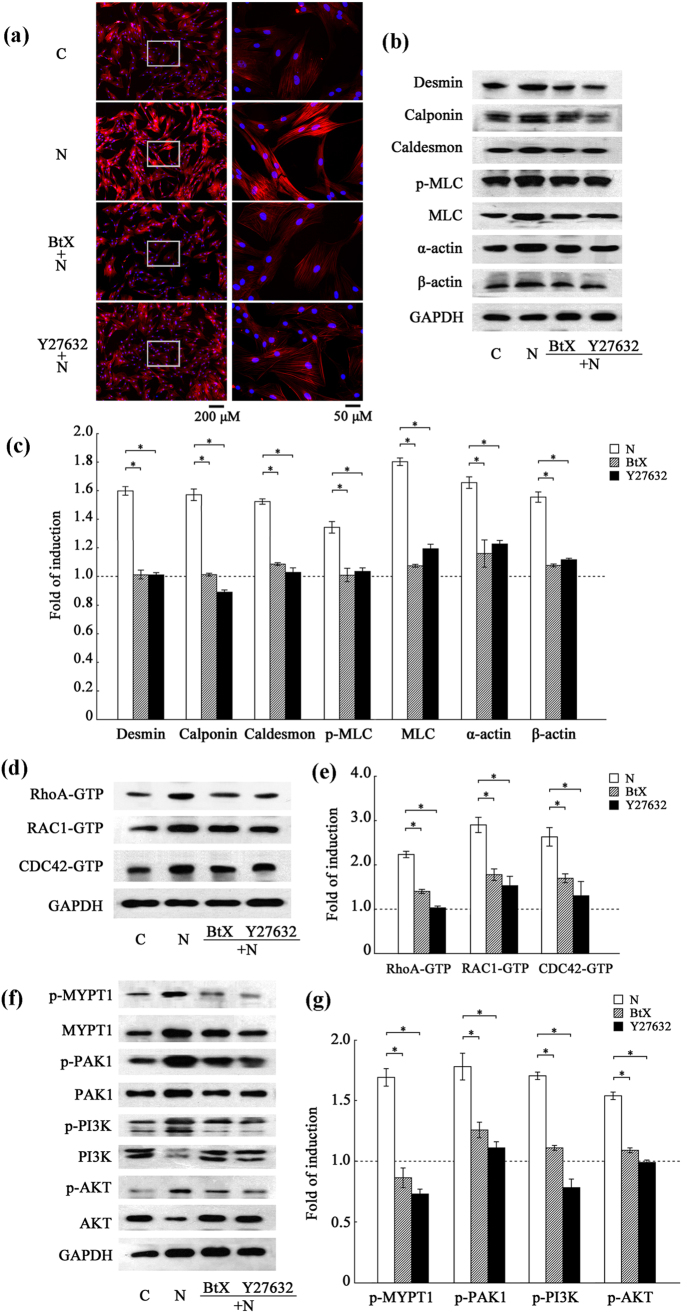
The Rho GTPase pathway is involved in the up-regulation of cytoskeleton-related proteins in nicotine-treated VSMCs. VSMCs were pretreated with α-BtX or Y27632 for 30 min and then exposed to nicotine for 24 h. (**a**) Fluorescent micrographs of the cells labeled with rhodamin-phalloidin for detection of F-actin. The right panel is the magnified image within the left rectangle. The nucleus was stained by DAPI. Representative images from three independent experiments were presented as red channel (F-actin), blue channel (DAPI) and merged figure. There was no signal detected when rhodamin-phalloidin was omitted (not shown). F-actin was up-regulated when VSMCs were treated with nicotine (N) compared to the control (C) and α-BtX or Y27632 pre-treatment counteracted this effect. (**b**) and (**c**) Western blot analysis of cytoskeletal proteins, differentiation markers and SM-specific contractile proteins (α-actin, β-actin, MLC, p-MLC, desmin, calponin and caldesmon). GAPDH was used as a reference gene. In the histogram, the fold change of the control (C) was standardized to 1 compared to the nicotine-treated group. The inhibitors attenuated the effect of nicotine by approximately 50% (*p < 0.05). The results are expressed as the mean ± SEM (n = 5). (**d**) and (**e**) Activation assay of Rho GTPases in VSMCs after nicotine treatment. The results showed that α-BtX or Y27632 inhibited the activities of Rho GTPases compared to the nicotine-treated group (N). The results are expressed as the mean ± SEM (*p < 0.05, n = 5). (**f**) and (**g**) Western blot analysis of the intracellular downstream signaling proteins of Rho GTPases after nicotine treatment. MYPT1 is a target of RhoA. PAK1 and PI3K/AKT are common targets of RAC and CDC42. GAPDH was used as an internal reference. The phosphorylation of MYPT1, PAK1 and PI3K/AKT represented the activation of RhoA, RAC1 and CDC42 signaling. The results showed that α-BtX or Y27632 inhibited the phosphorylation of Rho-GTPase downstream effectors compared with the nicotine-treated group (N) (*p < 0.05). The results are expressed as the mean ± SEM (n = 5).

Myosin, together with the dynamic actin filament, plays a crucial role in the proliferating and migrating cells and desmin, calponin and caldesmon have been regarded as SMC-special markers for their association with the actin filament, so eight cytoskeletal markers (α-actin, β-actin, F-actin, myosin light chain (MLC), phosphorylated myosin light chain (p-MLC), desmin, calponin and caldesmon) were detected in the study. Fluorescent micrographs (Fig. 1a) revealed that F-actin was up-regulated when VSMCs were treated with nicotine (N) compared to the control (C) and α-BtX or Y27632 pre-treatment counteracted this effect. This result indicates that the Rho GTPase pathway is involved in the change of F-actin expression induced by nicotine. Figure 1b showed the expression level of other cytoskeleton-related proteins (α-actin, β-actin, MLC, p-MLC, desmin, calponin and caldesmon). In the histogram (Fig. 1c), the control (C) was standardized to 1-fold induction. It was determined that treatment with nicotine (N) substantially increased expression of cytoskeleton-related proteins and addition of either α-BtX or Y27632 inhibited the considerable increase in cytoskeletal protein expression induced by nicotine (Fig. 1b,c).

To further understand the influence of nicotine on the Rho GTPase pathway, we examined the activation of the main Rho GTPases, namely, RhoA-GTP, RAC1-GTP and CDC42-GTP (Fig. 1d and e) as well as the downstream intracellular signaling proteins, including myosin phosphatase target subunit-1 (MYPT1), p21-activated kinase-1 (PAK1) and phosphatidylinositde-3 kinase/protein kinase B (PI3K/AKT) (Fig. 1f and g). Upon treatment with nicotine, there was an obvious activation of the Rho GTPase pathway and the downstream signaling pathway in VSMCs and both α-BtX and Y27632 could substantially inhibit this increased activation.

These results indicate that treatment with nicotine can activate the Rho GTPase pathway and induce the synthesis of the cytoskeletal proteins (α-actin, β-actin, F-actin, MLC, p-MLC, desmin, calponin and caldesmon) in VSMCs through the activation of intracellular downstream signaling pathways, including targets such as MYPT1, PAK1 and PI3K/AKT.

### The Rho GTPase pathway is associated with nicotine-induced migration and proliferation in VSMCs

**Figure 2:**
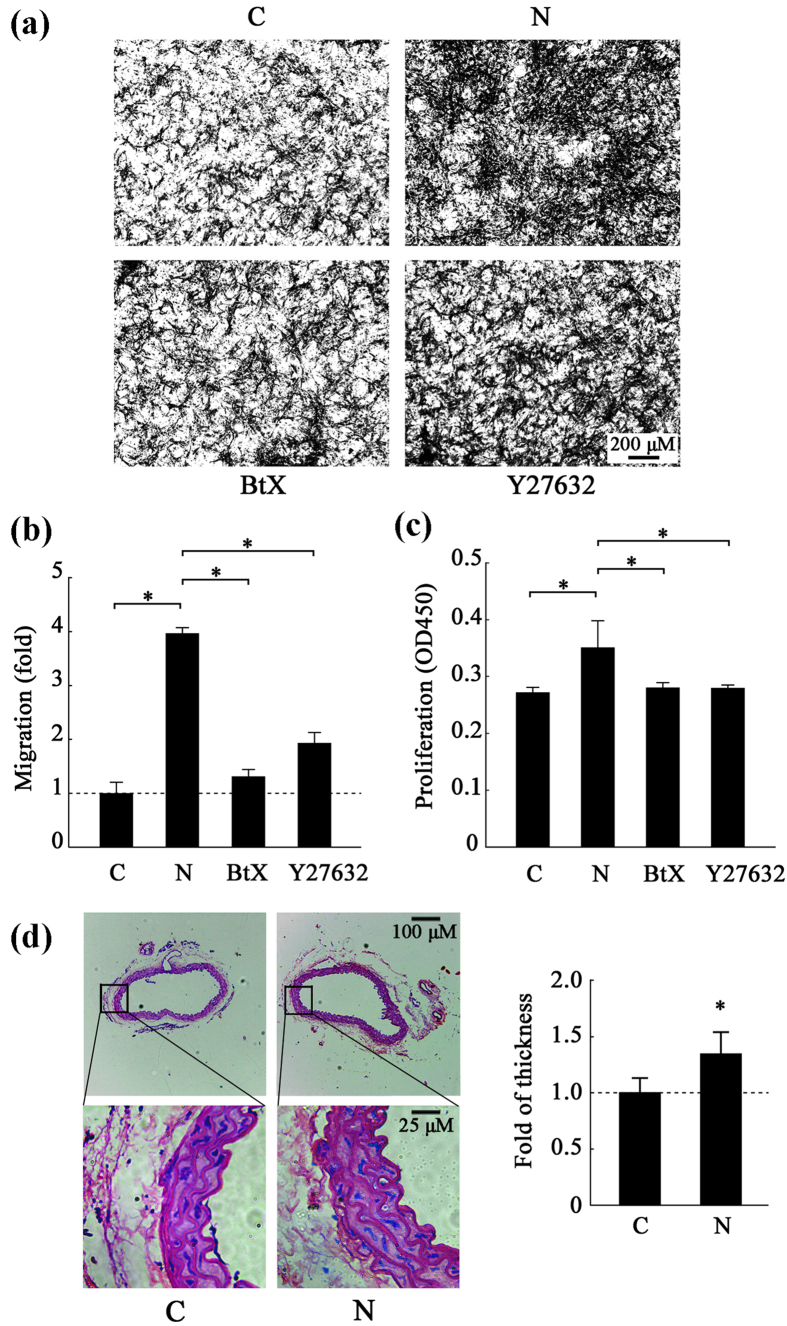
The role of the Rho GTPase pathway in the migration and proliferation of nicotine-induced VSMCs. (**a**) Migration images. The number of cells, which is a measure of migration ability, was detected by a transwell migration assay Representative images at a magnification of 50X from three independent experiments are presented. (**b**) A histogram of the transwell assay results. (**c**) A histogram of the CCK8 assay results. The results are expressed as the mean ± SEM (*p < 0.05, n = 5). (**d**) Hematoxylin-eosin staining of the thoracic arteries from PBS- and nicotine-treated mice and a histogram of the median thickness of the thoracic arteries. The values are expressed as the mean ± SEM (*p < 0.05, n = 20 mice per group × 3 times).

The ability of VSMCs to migrate *in vitro* was measured using an 8 μm-pore size transwell system and the proliferation rate was determined using a CCK8 assay. VSMCs were pretreated with α-BtX or Y27632 before a 24 h exposure to nicotine. In the histogram, the control (C) was standardized to 1-fold induction. Nicotine exposure stimulated a 3.6-fold increase in migration in the VSMCs compared to the control group and this increase was suppressed by 69% and 52% following treatment with α-BtX or Y27632, respectively (Fig. 2a and b). The CCK8 assay (Fig. 2c) showed an increase in the proliferation rate of VSMCs treated with nicotine; however, this increase could be suppressed by 23% and by 24% when the cells were pre-treated with α-BtX or Y27632, respectively.

To investigate the effect of nicotine on VSMCs *in vivo*, C57BL/6J mice were subjected to a treatment of nicotine for 20 weeks to mimic a long-term heavy smoking. As shown by hematoxylin-eosin staining (Fig. 2d), nicotine treatment significantly increased the median thickness of the thoracic arteries of the treated mice compared to the control group (p = 0.022). Meanwhile, the measurements for changes on vascular function (Supplementary Fig. S3) showed that nicotine had only tiny influence on blood pressure and heart rate; however, there is no significant difference between two groups (p < 0.05).

These results indicate that nicotine can induce increased migration and proliferation of VSMCs both *in vitro* and *in vivo* in a Rho GTPase-dependent manner.

### Upon nicotine treatment, the mRNA expression level of RhoGDIA is increased but protein level is decreased both *in vitro* and *in vivo*

**Figure 3:**
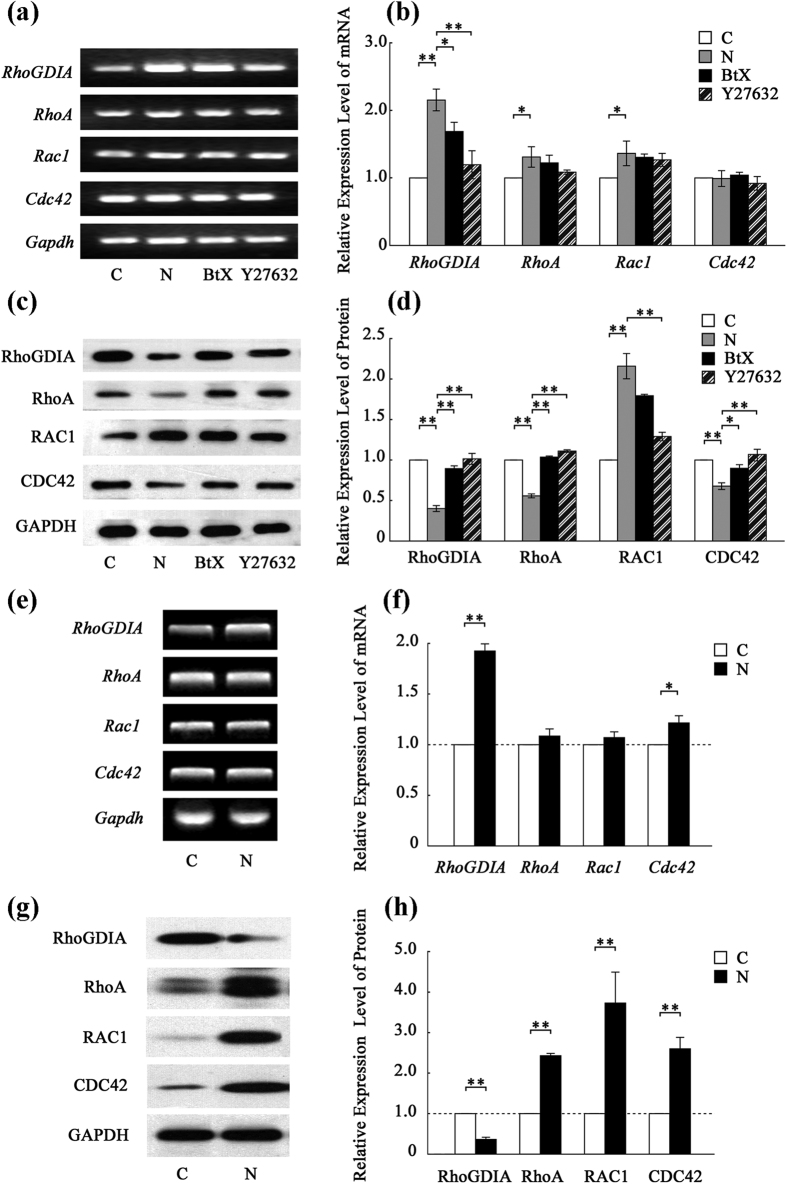
Detection of the expression levels of genes involved in the Rho GTPase pathway *in vitro* and *in vivo*. (**a**) qRT-PCR analysis of the target gene transcripts *in vitro*. The results are shown in histogram (**b**). (**c**) Western blots of target proteins *in vitro*. The results are shown in histogram (**d**). (**e**) Semi-quantitative reverse transcription PCR analysis of target gene transcripts *in vivo*. The results are shown in histogram (**f**). (**g**) Western blots of target proteins *in vivo*. The results are shown in histogram (**h**). The values are expressed as the mean ± SEM (n = 5, *p < 0.05, **p < 0.01).

To explore the molecular mechanisms underlying nicotine-induced Rho-GTPase-dependent migration and proliferation of VSMCs, the expression levels of Rho GTPase-related genes were detected. As shown by qRT-PCR analysis, among the four main members of the Rho family of GTPases (*RhoA, Rac1, Cdc42* and the regulatory factor *RhoGDIA*), *RhoGDIA* expression was seriously increased in response to nicotine treatment both *in vitro* (Fig. 3a and b) and *in vivo* (Fig. 3e and f). Furthermore, RhoGDIA protein expression was also evaluated. *In vitro*, treatment with nicotine decreased RhoGDIA expression by 42% compared to the control and this decrease was severely suppressed by α-BtX or Y27632 (Fig. 3c and d). Similar results were observed by Western blotting (Fig. 3g and h) and immunohistochemical staining of RhoGDIA *in vivo* (Supplementary Fig. S4).

In addition to the changes in RhoGDIA, there are also changes in mRNA and protein expression of total RhoA, RAC1 and CDC42, which hinted at more than one mechanism involved in the nicotine-induced Rho GTPase changes. Considering the abnormal co-activation of Rho GTPases and the function of RhoGDIA as a negative regulator of the Rho GTPase pathway, we thought that RhoGDIA may play an important role in the dysfunctions of VSMCs, so we focused on RhoGDIA and the mechanism underpinning its deregulation in the study.

### MiR-200b down-regulates *RhoGDIA* expression by targeting its 3′-UTR

**Figure 4:**
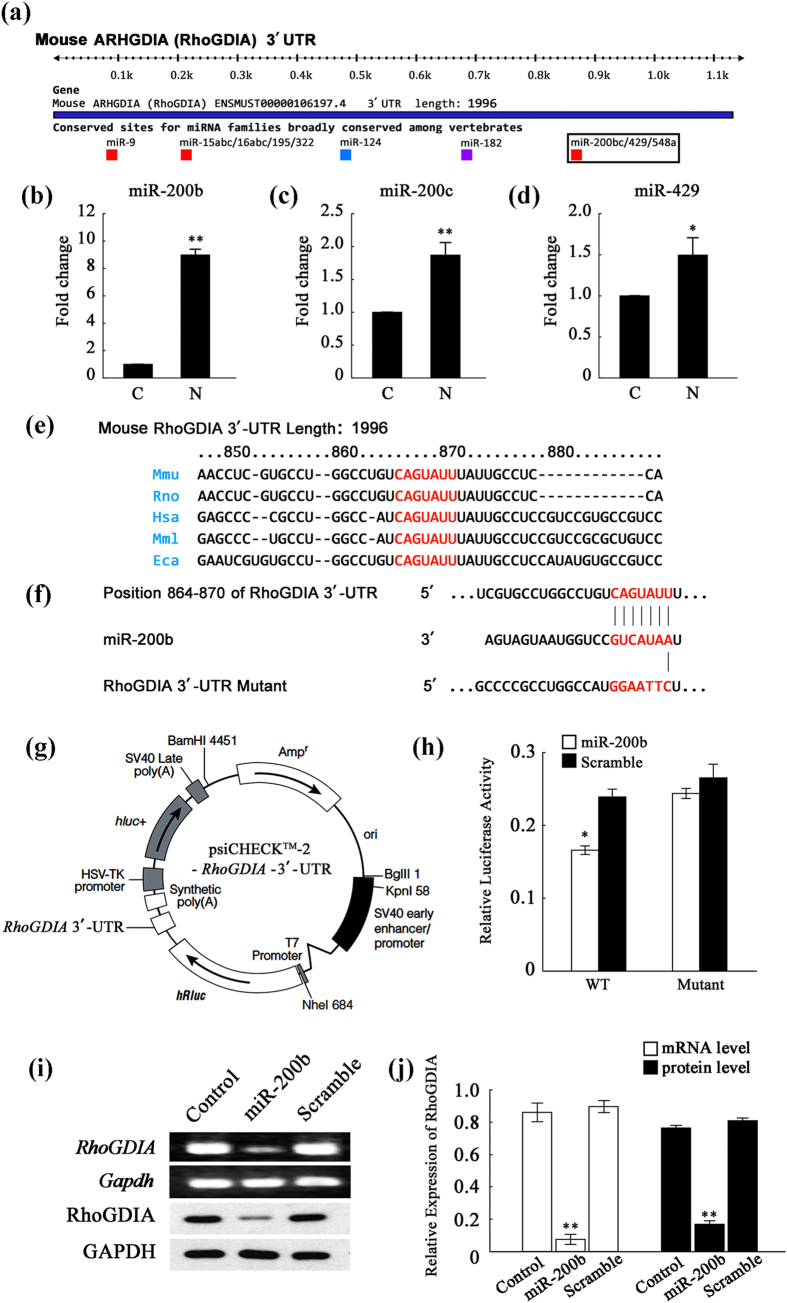
MiR-200b down-regulates the expression level of *RhoGDIA* mRNA. (**a**) Diagrammatic sketch of the 3′-UTR region of the mouse *RhoGDIA* gene. The miR-200b microRNA family, including miR-200b, miR-200c and miR-429, was predicted to bind the *RhoGDIA* 3′-UTR region. (**b–d**) Real-time PCR results regarding the expression levels of miR-200b, miR-200c and miR-429 in VSMCs. U6 (U6 small nuclear RNA) was used as an internal control. The results are expressed as the mean ± SEM (n = 5, *p < 0.05). (**e**) The nucleotide sequences of *RhoGDIA* from different species. (**f**) The nucleotide sequences of miR-200b and the complementary region of the *RhoGDIA* 3′-UTR. The mutant *RhoGDIA* 3′-UTR was constructed via point mutations of 6 nucleotides in the middle of the complementary region. The red letters illustrate the sequence alignment of miR-200b, the RhoGDIA 3′-UTR and the mutant RhoGDIA 3′-UTR. (**g**) The plasmid profile of the constructed dual-luciferase reporter vector psiCHECK^TM^-2-*RhoGDIA*-3′-UTR. (**h**) Luciferase activity reflecting that RhoGDIA expression in HeLa cells was suppressed by miR-200b but not by negative control (NC) miRNA. This inhibitory action of miR-200b was abrogated when the target sites of the *RhoGDIA* 3′-UTR were mutated (*p < 0.05). The results are expressed as the mean ± SEM (n = 5). (**i**) PCR and Western blot analysis of RhoGDIA after transfection of miR-200b into VSMCs. As shown in histogram j, miR-200b transfection sharply decreased RhoGDIA expression at the mRNA and protein levels (**p < 0.01). The values are expressed as the mean ± SEM (n = 5).

The expression of RhoGDIA can be regulated pre- and post-transcriptionally by epigenetic modifications of chromosomal DNAs and microRNAs. After examining the promoter region of *RhoGDIA*, we found no difference in methylation between the control and nicotine-treated groups (Supplementary Fig. S5). Nevertheless, the discrepancy observed between RhoGDIA mRNA and protein expression indicated that post-transcriptional regulation might be occurring. Consequently, microRNAs associated with RhoGDIA were identified. To predict the miRNAs that may regulate RhoGDIA expression, we used two of the most commonly utilized algorithms, TargetScan and miRWalk. A total of approximately 50 microRNAs were predicted to target *RhoGDIA*, most of which were found to have conserved seed sequences in the 3′-UTR of *RhoGDIA* (Fig. 4a). Interestingly, among these 50 microRNAs, three members of the miR-200 family, namely miR-200b, miR-200c and miR-429, have been confirmed to exist in VSMCs and are also implicated in the regulation of the cytoskeleton. Then, real-time PCR was performed to investigate the expression of miR-200b (Fig. 4b), miR-200c (Fig. 4c), and miR-429 (Fig. 4d). The results showed that nicotine treatment up-regulated the three miRNAs to different degrees, with a considerable increase in miR-200b expression.

Because miR-200b was elevated after nicotine treatment and at the same time its putative target gene *RhoGDIA* was down-regulated, a dual-luciferase reporter assay was conducted in HeLa cells to verify the regulatory relationship between miR-200b and *RhoGDIA*. The 3′-UTR of *RhoGDIA* was inserted into the psiCHECK^TM^-2 luciferase reporter vector and a mutant of the 3′-UTR reporter was also constructed by mutating 6 nucleotides (Fig. 4e,f and g). The results indicated that miR-200b significantly inhibited luciferase activity and this inhibitory effect was lost in the mutant vector (Fig. 4h, p < 0.001). Furthermore, miR-200b mimics were directly transfected into the VSMCs, which endogenously express RhoGDIA. As shown in Fig. 4i and j, the mRNA and protein levels of RhoGDIA were severely attenuated in the VSMCs transfected with the miR-200b mimics, while the scrambled RNA failed to inhibit RhoGDIA expression. These results verify that *RhoGDIA* is a target gene of miR-200b.

### Nicotine up-regulates miR-200b expression through promoter hypomethylation

**Figure 5:**
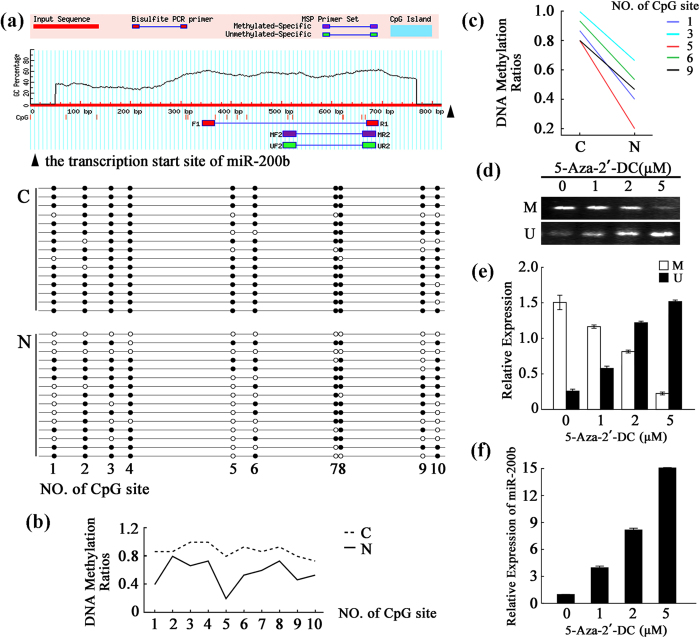
Up-regulation of *miR-200b* was associated with hypomethylation of DNA in the promoter region in nicotine-treated VSMCs. (**a**) CpG methylation in the control and nicotine-treated VSMCs. CpG islands were predicted and bisulfite-sequencing PCR primers were designed using the Methprimer tool. The CpG islands were tinted blue and the BSP primers flanked a 350-bp PCR product upstream of the transcription start site of *miR-200b*. Each spot indicates one methylation site (CpG); the black spots indicate methylated cytosines, and the white spots indicate unmethylated cytosines. (**b**) A line graph of the DNA methylation ratios in CpG sites 1–10 from control and nicotine-treated VSMCs. (**c**) A histogram of the DNA methylation ratios in CpG sites 1, 3, 5, 6 and 9 in control and nicotine-treated VSMCs. (**d–f**) Verification of DNA methylation regulation in VSMCs. The panels (**d**) show the MSP results for the primers that were designed for the methylated and unmethylated sites in VSMCs that were treated with a gradient of 5-Aza-2′-DC concentrations. The results are shown in histogram (**e**). Histogram f shows the results of the real-time PCR analysis of the *miR-200b* expression level in VSMCs treated with 5-Aza-2′-DC. The values are expressed as the mean ± SEM (n = 5).

To further explore the molecular mechanism responsible for nicotine-induced up-regulation of miR-200b, the level of the primary miR-200b transcript (pri-miR-200b) was measured (Supplementary Fig. S6). The result revealed a significant increase in pri-miR-200b in the nicotine-treated VSMCs (p < 0.001). Then, the promoter region of *miR-200b* was analyzed. Although the promoter lacked CpG islands, the enrichment of CpG sites in the CpG island shore region (Fig. 5a) indicated that *miR-200b* might be an epigenetically modified gene. Thus, the DNA methylation pattern was investigated by designing primers that flanked a 350-bp fragment and utilizing bisulfite sequencing analysis via MethPrimer. The sequencing information confirmed the presence of 10 CpG sites. The methylation level (the percentage of methylated-CpGs compared to the total number of CpGs) was 88.0% in the control group and this level was decreased to 56.7% in the nicotine-treated group (Fig. 5a and b). Moreover, methylation occurred less frequently at sites 1, 3, 5, 6 and 9 (Fig. 5c). The results demonstrate that *miR-200b* undergoes epigenetic changes following nicotine treatment.

To confirm the epigenetic regulation of *miR-200b*, 5-aza-2′-deoxycytidine (5-Aza-2′-DC) was used in VSMCs. Following methylation-specific and non-methylation-specific-PCR (Fig. 5d and e), low doses of 5-Aza-2′-DC were able to change the methylation status of the *miR-200b* promoter. This change was accompanied by an obvious increase in the level of *miR-200b* (Fig. 5f). These results confirm that miR-200b is dose-dependently elevated by treatment with 5-Aza-2′-DC, which attenuates the methylation level of the CpG island shore region of *miR-200b*.

Meanwhile, the real-time PCR results showed a significant increase of miR-200b expression in the thoracic arteries of nicotine-treated mice compared to the control group (Supplementary Fig. S7a, p < 0.001). A bisulfite sequencing analysis of the promoter region of *miR-200b* was also performed on the thoracic arteries of nicotine-treated mice (Supplementary Fig. S7b). The hypomethylation that was observed demonstrated that *miR-200b* underwent epigenetic changes following nicotine treatment *in vivo*. These results suggest that nicotine can up-regulate miR-200b expression through promoter hypomethylation.

### The miR-200b-mediated increase in the activities of RhoA, RAC1 and CDC42 facilitates migration and proliferation of VSMCs treated with nicotine

**Figure 6:**
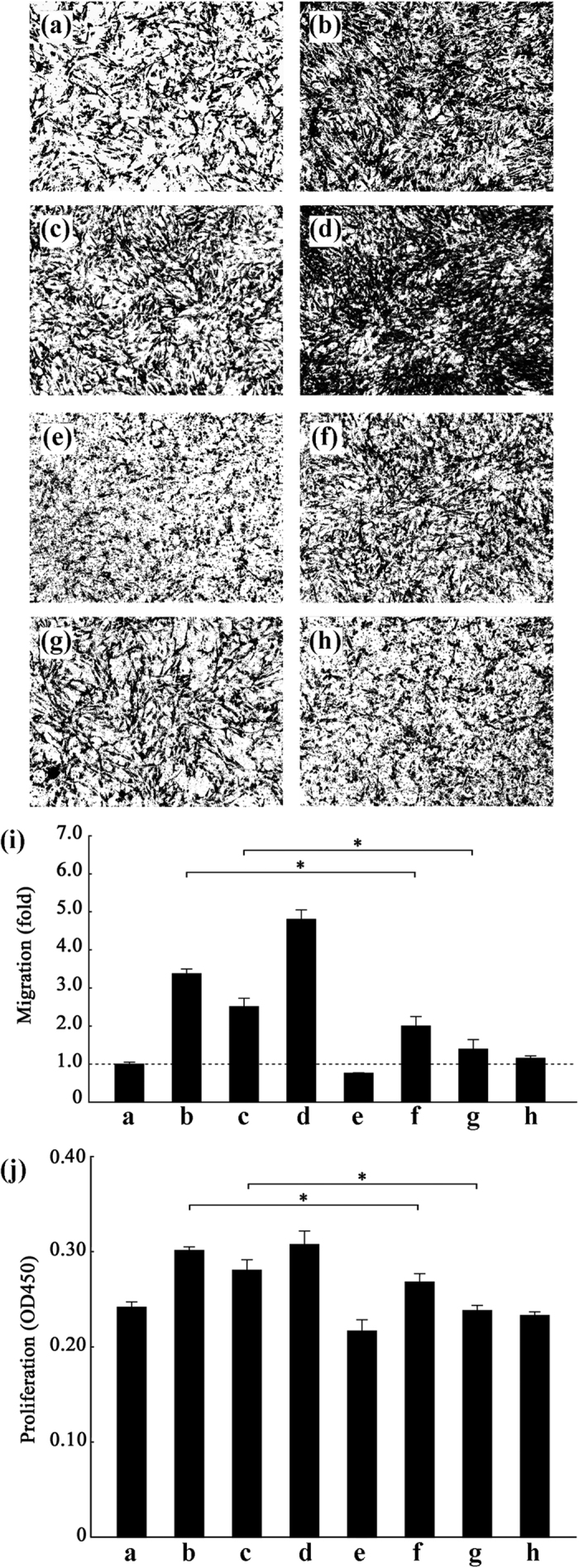
MiR-200b facilitates the migration and proliferation of VSMCs. (**a–h**) Migration images. The number of cells, which is a measure of migration rate, was detected by a transwell migration assay Representative images at a magnification of 50X from three independent experiments are presented. The results are shown in histogram (**i**). (**a**) the control group; (**b**) VSMCs exposed to 10 μM nicotine for 24 h; (**c**) VSMCs transfected with miR-200b mimics (100 nmol/L); (**d**) VSMCs treated with miR-200b mimics (100 nmol/L) and nicotine; (**e**) VSMCs transfected with miR-200b (**f**) VSMCs treated with both miR-200b inhibitors (100 nmol/L) and nicotine; (**g**) VSMCs co-transfected with both miR-200b mimics and miR-200b inhibitors (100 nmol/L); (**h**) VSMCs transfected with scrambled miRNAs (100 nmol/L). The image of VSMCs treated with nicotine, miR-200b mimics (100 nmol/L) and miR-200b inhibitors (100 nmol/L) is shown in Supplementary Fig. S9. (**j**) A histogram of the CCK8 assay results from the eight groups. The values are expressed as the mean ± SEM (n = 5, *p < 0.05).

To determine whether miR-200b played a crucial role in the dysfunction of VSMCs treated with nicotine, we measured migration and proliferation of VSMCs transfected with miR-200b mimics or inhibitors (Fig. 6). Transfection with miR-200b mimics induced migration and proliferation in VSMCs. Furthermore, miR-200b inhibitors depressed migration and proliferation of VSMCs that were treated with either nicotine or miR-200b mimics.

Given that miR-200b can promote dysfunction in VSMCs, we examined the activation of the main Rho GTPases after transfecting VSMCs with miR-200b mimics. Cells transfected with miR-200b had a sharp decrease in RhoGDIA expression and this effect in turn activated RhoA, RAC1 and CDC42 (Supplementary Fig. S8). Taken together, these findings suggest that miR-200b promotes the dysfunction VSMCs mainly through the targeting of RhoGDIA, which is a well-known negative regulator of Rho GTPases.

In conclusion, our results demonstrate that nicotine treatment up-regulates miR-200b expression due to hypomethylation in the promoter region of *miR-200b* in VSMCs. MiR-200b-mediated down-regulation of RhoGDIA increases the activities of Rho GTPases to facilitate the migration and proliferation of VSMCs.

## Discussion

In the present study, we investigated the function of the Rho GTPase pathway underlying nicotine-induced abnormal behaviors in VSMCs. The results reveal that nicotine treatment significantly increases miR-200b expression through promoter hypomethylation. The miR-200b-mediated down-regulation of RhoGDIA in turn facilitates migration and proliferation of VSMCs in a Rho GTPase-dependent manner.

First, we examined the expression levels of a large panel of cytoskeletal proteins and SM-specific contractile proteins, including α-actin, β-actin, F-actin, MLC, p-MLC, desmin, calponin and caldesmon. As described previously, nicotine can induce the synthesis of cytoskeleton-related proteins^34^. Meanwhile, this induction can be attenuated by the Rho GTPase pathway inhibitor Y27632.

In addition, our results demonstrate that after nicotine treatment, the Rho GTPase pathway is aberrantly activated, which activates downstream targets such as MYPT1 (a target of RhoA), PAK1 (a common target of RAC and CDC42), and PI3K (which is bound and stimulated by both RAC and CDC42) in VSMCs. The mammalian family of Rho GTPases mainly consists of three subfamilies, Rho (RhoA, RhoB and RhoC), RAC (RAC1, RAC2 and RAC3) and CDC42 (CDC42Hs and G25K). At any given time in the cell, only small fractions of the Rho GTPases are associated with membranes and in the active state. Rho GTPases are maintained in the inactive state in the cytosol through association with RhoGDIs^12^,^35^. Our results show that the proportion of active Rho GTPases (namely RhoA-GTP, RAC1-GTP and CDC42-GTP) is increased in response to nicotine and RhoGDIA plays a crucial role in the process. These results provide new insights into the understanding of nicotine-induced dysfunction of VSMCs: Rho activation leads to the assembly of contractile actin-myosin filaments; in other words, Rho activation functions as a molecular switch, controlling signal transduction pathways that links the membrane receptors to the cytoskeleton^13^. For example, Rho A activates ROCK1 and ROCK2 in VSMCs^36^ and ROCK can directly phosphorylate myosin light chains *in vitro* at Ser19^37^. Additionally, PAK has also been reported to phosphorylate myosin light chains directly, which would activate actomyosin^38^. Consequently, when VSMCs are exposed to nicotine, the Rho GTPase pathway is activated, resulting in the persistent transcription of downstream genes through the phosphorylation of Rho effectors. As a result, the migration and proliferation rate of VSMS is enhanced. Previous studies^39^ have shown that contractile VSMCs, which are predominant in normal media, can be induced by nicotine to transform into synthetic VSMCs. Consistent with this, our study suggests that nicotine induces a number of cytoskeletal proteins and SM-specific contractile proteins (in particular, MLC, p-MLC, caldesmon, calponin and desmin) via the Rho GTPase pathway.

In the study, we also identified, for the first time, RhoGDIA as a direct and functional target of miR-200b. The results showed that miR-200b played a critical role in targeting RhoGDIA and thus activating Rho in the nicotine-treated VSMCs. Another important finding in our current study was that miR-200b up-regulation in VSMCs was due to hypomethylation of its neighboring CpG islands. It has been reported that DNA methylation plays an important role in regulating miRNA expression during tumorigenesis and cardiovascular diseases^40^,^41^. Aberrant DNA methylation of gene promoters is a common mechanism of miRNA deregulation at the transcriptional level^42^,^43^. Our findings demonstrate that the hypomethylation of upstream DNA CpG sites is closely related to miR-200b up-regulation and VSMC dysfunction induced by nicotine.

**Figure 7:**
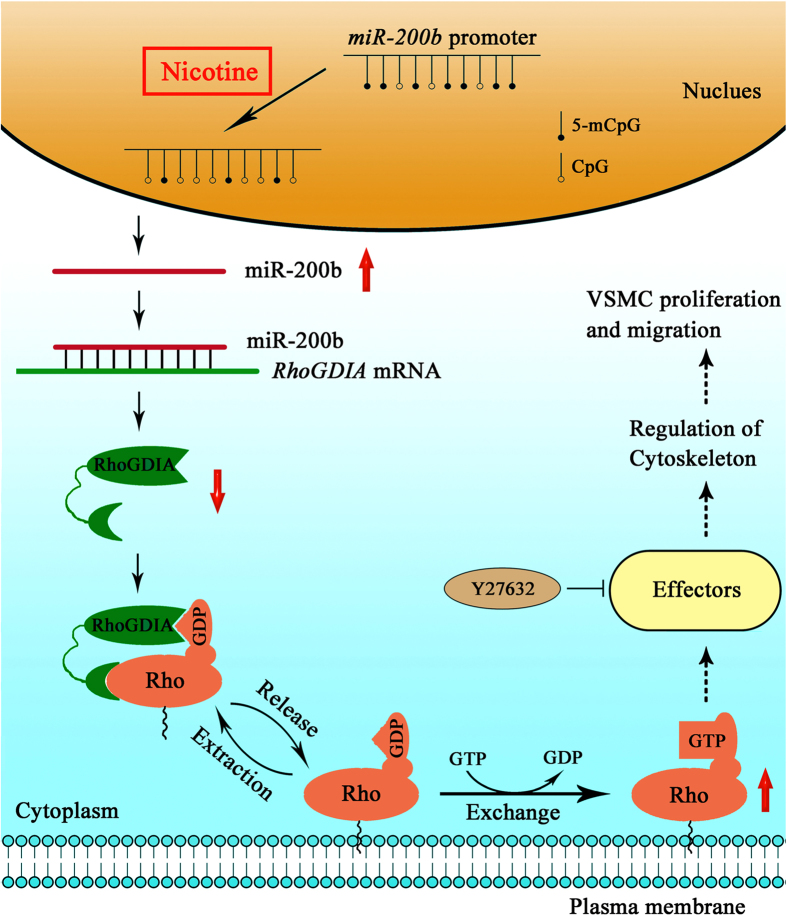
A diagram representing the mechanisms underlying nicotine-induced dysfunction in VSMCs.

As summarized in Fig. 7, our study provides strong evidence to explain the mechanisms underlying nicotine-induced dysfunction in VSMCs. It is worth noting that epigenetic modifications may play an important role in this pathological progression.

Additionally, it is interesting that nicotine can upregulate the transcription of RhoGDIA and miR-200b in VSMCs, but when given miR-200b to VSMCs *in vitro*, it can suppress RhoGDIA mRNA level. The cause of the discrepancy might be that nicotine could influence the expression of RhoGDIA mRNA via a variety of signaling molecules and pathways, while miR-200b affects the expression of RhoGDIA mRNA by directly targeting RhoGDIA. The observations imply that an intracellular negative feedback loop may contribute to the persistent transcription of *RhoGDIA* upon nicotine treatment. Further studies may be required to definitively demonstrate this idea. Meanwhile, we found considerable changes in the level of total RhoA and RAC1 mRNA and protein expression as well as in total CDC42 protein expression in the *in vitro* experiments. Major differences were also observed between the *in vitro* and *in vivo* experiments. The level of total RhoA and CDC42 protein was increased *in vivo* but decreased *in vitro* upon nicotine treatment. The inconsistency may be due to the difference in the level of nicotine treatment in the *in vitro* and *in vivo* experiments. *In vitro*, we used an experimental concentration of 10 μM, which is consistent with the nicotine concentration (10^−5^ to 10^−8^ M) that was determined from plasma isolated from smokers^33^. Meanwhile, mice were treated with 0.2 mg/100 g free-base nicotine *in vivo* according to the definition of “heavy smoking” (a consumption ≥ 20 cigarettes daily) by the World Health Organization (WHO)^44^,^45^. Since the mechanism of vascular senescence in aging mice is complex, mice were treated with nicotine for 20 weeks after sexually maturation to mimic nicotine-exposure at middle age. On the other hand, these *in vitro* and *in vivo* observations strongly implied more than one mechanism is involved in aberrant Rho GTPase expression as a result of nicotine exposure. As for other involved mechanisms, we speculate that in nicotine-treated cells, miR-200b may function in conjunction with other miRNAs such as miR-155. Taking RhoA as an example, we found no difference in the methylation pattern of the promoter region in the control and nicotine-treated groups (Supplementary Fig. S10a). However, miRNA-155, which has been identified to target RhoA in VSMCs^46^, was up-regulated after nicotine exposure (Supplementary Fig. S10b). This might explain why we observed a down-regulation in total RhoA protein level following nicotine treatment *in vitro*. The regulatory network of miRNAs is complicated, since a single miRNA can regulate multiple targets and a target can be regulated by multiple miRNAs. Furthermore, miR-200b has been reported to be linked to the down-regulation of total RhoA. Thus, further studies are required to understand the mechanisms underlying nicotine-induced changes in total RhoA, along with total RAC1 and total CDC42.

In addition to the Rho GTPase pathway, other pathways have been shown to be involved in the dysfunction VSMCs. A study by Wang *et al*. established that nicotine can trigger AMPK/AP2 signaling, resulting in aberrant expression of MMPs and consequent degradation of the extracellular matrix in VSMCs^4^. Other reports have shown that NF-κB pathway^34^, EGFR/ERK pathway^47^ and MAPK pathway^5^ also contribute to the dysfunction of VSMCs induced by nicotine. In our study, nicotine-induced proliferation is abrogated by prior treatment with Y27632, while the increase in migration was only partially prevented by Y27632, suggesting the involvement of different pathways in nicotine-mediated effects. Nevertheless, our results provide evidence that nicotine induces the dysfunction of VSMCs by affecting miR-200b, RhoGDIA, and the cytoskeleton.

In summary, our results demonstrated that nicotine treatment significantly increases miR-200b expression through promoter hypomethylation. The miR-200b-mediated down-regulation of RhoGDIA in turn facilitates migration and proliferation of VSMCs in a Rho GTPase-dependent manner. This newly identified mechanism involving miR-200b and RhoGDIA provides a new avenue in understanding the process of VSMC migration and proliferation and may facilitate the development of potential therapeutic agents for cardiovascular diseases.

## Materials and Methods

### Materials

Nicotine, Y27632, and α-bungarotoxin (α-Btx) were purchased from Sigma–Aldrich (St Louis, MO, USA). Antibodies against RhoGDIA (10509-1), RhoA (10749-1), RAC1 (24072-1), CDC42 (10155-1), MLC (10906-1), PAK1 (21401-1), MYPT1 (22117-1), desmin (16520-1), calponin (24855-1), and caldesmon (20887-1) were obtained from Proteintech Group Incorporation (Chicago, IL, USA). Actin (sc-10731), p-MYPT1 (sc-17432), p-PAK1 (sc-31685), p-MLC (sc-12896) and GAPDH (sc-25778) antibodies were obtained from Santa Cruz Biotechnology (Santa Cruz, CA, USA). An antibody against Alpha-Actin (#1184-1) was obtained from Epitomics (Burlingame, CA, USA). The Rho and Rac1/Cdc42 activation assay biochem kits were purchased from Cytoskeleton Incorporation (Denver, IL, USA). Other chemicals were of the highest grade commercially available.

### Cell culture

Mouse primary VSMCs were obtained from the thoracic arteries of mice using the cell explant method and were then propagated in high-glucose Dulbecco’s Modified Eagle’s Medium (DMEM) with 10% heat-inactivated fetal bovine serum (FBS; purchased from Gibco-BRL, Carlsbad, CA, USA), 50 IU/ml of penicillin and 50 IU/ml of streptomycin in a humidified incubator containing 5% CO_2_ at 37 °C. The VSMCs were characterized by the typical “hill-and-valley” morphology and had positive immunostaining for smooth muscle-specific α-actin. Cells between passages 2 and 4 were used for subsequent experiments and the experiments were performed on cells coming from different batches.

The HeLa cell line was cultured in DMEM media supplemented with 50 IU/ml penicillin, 50 IU/ml streptomycin and 10% FBS under 5% CO2 at 37 °C.

### Western blot analysis and Rho GTPase activation assays

VSMC proteins from differently treated groups of cells were extracted using TRIzol reagent and were subjected to SDS-polyacrylamide gel (SDS-PAGE) and electrophoretically transferred to PVDF membranes. The membranes were blocked at room temperature for 1 h with 5% fat- free milk in TBS containing 0.1% Tween 20 (TBST) and incubated with primary antibodies against RhoGDIA, RhoA, RAC1, CDC42, Desmin, Calponin, Caldesmon, β-actin, α-actin, MLC, p-MLC, PAK1, p-PAK1, MYPT1, p-MYPT1, PI3K, p-PI3K, AKT, p-AKT or GAPDH at 4 °C overnight. After being washed three times in TBST, the membrane was incubated with HRP-conjugated secondary antibody for 1 h at room temperature and then washed again. Finally, membranes were developed with an ECL detection reagent and exposed to X-ray film. The relative density of the protein bands was analyzed by ImageJ software and normalized to the respective GAPDH bands.

The activation of Rho GTPases was determined with Rho and RAC1/CDC42 activation assay kits in accordance with the manufacturer’s instructions. An analysis was performed with SDS-PAGE and Western blotting with anti-RhoA (1:2000), anti-RAC1 (1:2000) and anti-CDC42 (1:2000) antibodies.

### Real-time PCR and Reverse Transcription PCR analysis

Total RNA was extracted from treated cells with TRIzol according to the manufacturer’s protocol. The quantity and quality were determined using a Beckman DU800 spectrophotometer. For the detection of mRNAs and mature miRNAs, all RNA samples were reverse-transcribed into cDNA using the SYBR^®^ PrimeScript™ RT-PCR Kit (TaKaRa, Otsu, Japan). Then, real-time PCR reactions were performed using an ABI PRISM 7500 (Applied Biosystems, USA) following the manufacturer’s protocols. U6 was used as an internal control for normalization of miRNA levels and GAPDH was used for normalizations of mRNA levels. Sequences of primers used in the analysis were shown in Supplementary Table 1. Relative quantification (RQ) was calculated using the 2^−Δ(ΔCt)^ method, where RQ or fold change is equal to 2^− ((Mean ΔCt Target) − (Mean ΔCt Calibrator))^.

### Dual-Luciferase Reporter Assay

Luciferase reporters (psiCHECK2-*RhoGDIA*-3′-UTR) were constructed by inserting the 3′-UTR fragment of the mouse *RhoGDIA* gene into a psiCHECK2 reporter vector (Promega) using the NotI/XhoI cut sites. Mutations in the 3′-UTR were generated by mutating 6 nucleotides in the wild-type (WT) construct of psiCHECK2- *RhoGDIA*-3′-UTR using the Takara MutanBEST Kit (Takara). The miR-200b mimic was synthesized by Shanghai GenePharma. HeLa cells were co-transfected with the luciferase reporter vectors and the miR-200b mimics or scrambled miRNAs using Lipofectamine 2000 reagent (Invitrogen). The luciferase activity of Firefly and Renilla was measured sequentially with a dual-luciferase assay (Promega) 24 h after transfection according to the manufacturer’s instructions.

### Bisulfite DNA Sequencing PCR (BSP) and Methylation Specific-PCR (MSP)

#### Analyses

Genomic DNA was isolated from cells using a DNeasy Tissue kit (Qiagen, Germany) after VSMCs were treated. Purified genomic DNA (1 μg) was treated with the Methylamp DNA Modification Kit (EPIGENTEK, USA). The eluted DNA (2 μl) was PCR amplified with miR-200b-specific bisulfate sequencing primers (miR-200b-BSP-F: 5′-TGGATTTGGAGGGTGAGTTATATAA-3′; and miR-200b-BSP-R: 5′- CCCTTTCCAAAATACCTTAATCCT-3′). The PCR product (350 bp) was restored and cloned into the pEASY-T5 vector (TaKaRa, Japan). After transformation into competent bacterial cells, 15 clones from each of the two groups were sequenced. The BiQ analyzer (http://biq-analyzer.bioinf.mpi-inf.mpg.de/) was used to analyze the sequencing results.

After VSMCs were cultured to 90–95% confluency, 5-aza-2′-deoxycytidine (5-Aza-2′-DC) (Sigma, Cat. No. A3656) was added to the culture media to a final concentration of 0, 1, 2 or 5 μM. The cells were harvested with TRIzol (Invitrogen, Carlsbad, CA, USA) 48 h after treatment. Genomic DNA that was isolated from the VSMCs was subsequently treated with the Methylamp DNA Modification Kit, and the eluted DNA (2 μl) was amplified with two sets of MSP primers (miR-200b-M-F: 5′-TTTTTTTTATAGTCGTTGGTATCGA-3′ and miR-200b-M-R: 5′-CTTTCCAAAATACCTTAATCCTCG-3′; miR-200b-U-F: 5′-TTTTTTTTATAGTTGTTGGTATTGA-3′ and miR-200b-U-R: 5′-CTTTCCAAAATACCTTAATCCTCAC-3′). The forward primer contains the 5th and 6th CpG sites. The reverse primer contains the 10th CpG site.

To ensure more specific PCR products in BSP and MSP assays, a touch-down PCR method with an annealing temperature from 60 °C to 55 °C and EX-Taq HS (TaKaRa, Japan) was used. The PCR products were resolved by electrophoresis with a 2% agarose gel.

### Cell migration Assay

VSMC migration study was performed using the transwell system (BD Transduction) with a pore size of 8 μm in 24-well plates, as described previously^49^. Briefly, VSMCs treated with nicotine or transfected with miRNAs were seeded at 1.0 × 10^6^ cells/ml in the upper chamber, and the lower chamber was filled with 600 μl DMEM with 50% heat-inactivated FBS to serve as a chemoattractant. After 24 h of attraction, the number of migrated cells was counted under a microscope (magnification 200×, Olympus). All of the cells in each transwell membrane were counted.

### Cell Proliferation Assay

Cells were plated in 96-well plates (100 μl, 4000 cells per well) overnight and then treated with nicotine or transfected with miRNAs. After 48 hours, cell counting kit-8 (10% v/v) was added for an additional 1 hour before absorbance was measured in quintuplicate for each sample with a microplate spectrophotometer reader at 450 nm.

### Animal Model and Nicotine Treatments

Forty 6-week-old male C57BL/6 J mice (acquired from Shanghai SLAC Laboratory Animal Co. Ltd.) were randomly divided into control and nicotine-treated groups of 20 mice each. The nicotine-treated group received a total of 0.2 mg/100 g of free-base nicotine via intraperitoneal injection to mimic the blood plasma levels of nicotine in heavy smokers (≥20 cigarettes/day)^50^. The mice received 4 (q.q.h. during the day) nicotine treatments per day; a low dose (0.05 mg/100 g) was given for each injection to avoid producing any malaise or sickness in the mice. The control group received an equivalent amount of saline solution.

Blood pressure was measured in the C57Bl/6J mouse by the tail-cuff method after 20 weeks of treatment. In brief, blood pressure was recorded in the unrestrained, conscious mice over 24 hours for 6 times. Moving averages of systolic blood pressure (SBP), diastolic blood pressure (DBP) and heart rate were obtained over 24 hours. Mean blood pressure was calculated as (SBP+2 DBP)/3 from the recorded data.

After 20 weeks of treatment and blood pressure measurement, the two groups of mice were sacrificed using cervical dislocation and thoracic artery samples were collected after PBS perfusion. Samples for further research were rinsed in PBS and stored at −80 °C.

### Approval Statement

The animal experiments were carried out in compliance with the Guide for the Care and Use of Laboratory Animals of the Ministry of Health of China and NIH and the Declaration of Helsinki. The studies have been approved by the Bioethics Committee of Shanghai Jiao Tong University, China (approval reference number: 2013-1-010).

### Immunohistochemistry

The blood vessel tissues isolated from the two groups of mice were immersed and embedded in paraffin. These 5-μm paraffin sections were then cut and mounted onto poly-L-lysine-coated glass slides. The sections were baked at 85 °C for 15 min and then deparaffinized according to the standard procedure. The rehydrated sections were washed and 0.01 M citrate buffer (pH 6.0) was used for antigen retrieval in a pressure-cooker. After the sections were naturally cooled in citrate buffer, they were treated with 1% Triton X-100 for 15 min and incubated with 5% BSA (in PBS, pH = 7.4) for 30 min. This procedure was followed by rinsing with TBS 3 times for 5 min and an overnight incubation with primary antibodies at 4 °C. After washing with PBS, the sections were incubated with the HRP-conjugated secondary antibody for 1 h, followed by DAB staining. Finally, the sections were dehydrated, cover-slipped and examined by microscopy.

### Bioinformatics and statistical analyses

The miRNAs linked with organs and diseases were explored in miRWalk (http://www.umm.uni-heidelberg.de/apps/zmf/mirwalk/index.html). All methprimers were designed using MethPrimers (http://www.urogene.org/cgi-bin/methprimer/methprimer.cgi).

All statistical computations were calculated using SPSS version 19. The independent experiments were performed at least in triplicate and one-way ANOVA was used to compare the results between the two groups followed by a Fisher’s t-test for multiple comparisons. A significant difference is indicated by “*” (p < 0.05) and an extremely significant difference is indicated by “**”(p < 0.01).

## Additional Information

**How to cite this article:** Liang, D. *et al*. Nicotine facilitates VSMC dysfunction through a miR-200b/RhoGDIA/cytoskeleton module. *Sci. Rep.* **7**, 43798; doi: 10.1038/srep43798 (2017).

**Publisher's note:** Springer Nature remains neutral with regard to jurisdictional claims in published maps and institutional affiliations.

## Supplementary Material

Supplementary Figures

Supplementary Dataset 1
