# Supplementary material for: Nicotine facilitates VSMC dysfunction through a miR-200b/RhoGDIA/cytoskeleton module
Source: Sci Rep. 2017 Mar 2;7:43798. doi: 10.1038/srep43798 (PMC5333142; doi:10.1038/srep43798)
Supplement: Supplementary Figures [file srep43798-s1.pdf]

Nicotine facilitates VSMC dysfunction through a miR-200b/RhoGDIA/cytoskeleton module

Dongli Liang[1, 4], Zhaoxia Wang[2, 4], Zhiqiang Yan[1], Shangwei Hou[1], Wangjie Xu[1], Lianyun Wang[1], Meisheng Shang[3, *], Zhongdong Qiao[1, **]

[1]School of Life Sciences and Biotechnology, Shanghai Jiao Tong University, Shanghai, 200240, PR China

[2] Laboratory Animal Center of Instrumental Analysis Center, Shanghai Jiao Tong University, Shanghai, 200240, PR China

[3]Beijing Anzhen Hospital, Capital Medical University, Beijing, 100029, PR China

[4] These authors contributed equally to this work

[**] Correspondence author, E-mail: zdqiao@sjtu.edu.cn

[*] Co-correspondence author, E-mail: sxtysms@163.com

School of Life Science and Biotechnology, Shanghai Jiao Tong University, Shanghai, P. R. China

Shanghai Jiao Tong University, 800 Dongchuan Road, Shanghai, 200240, P. R. China

Tel.: +86 21 34204925

Fax: +86 21 54747330

Supplementary Figure 1. The expression of RhoGDIA at different concentrations of nicotine.

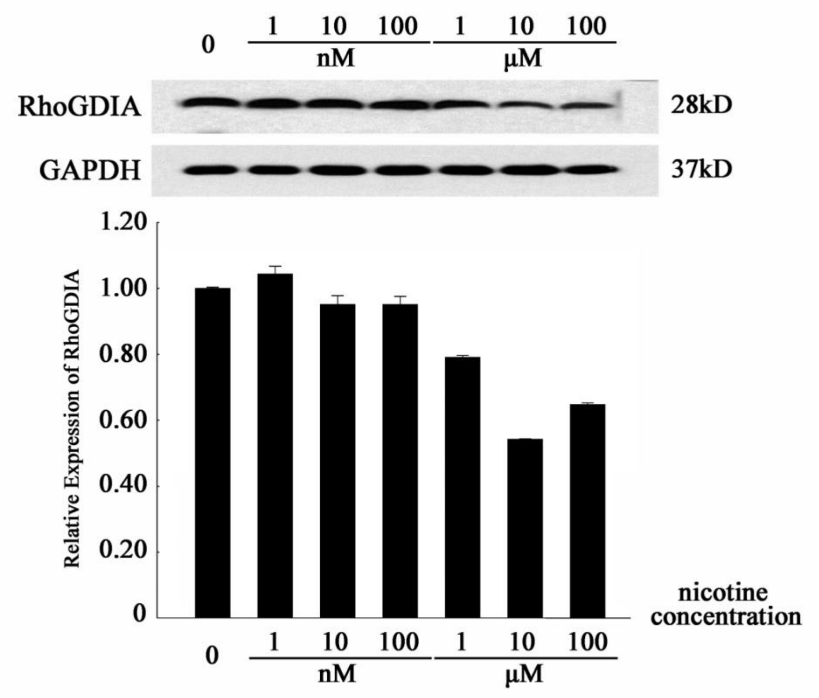

VSMCs were incubated with different concentrations of nicotine (10^{-9}, 10^{-8}, 10^{-7}, 10^{-6}, 10^{-5}, 10^{-4}M) for 24 h. The expression of RhoGDIA was detected by Western blotting. The values are expressed as the mean ± SEM (n=5).

Supplementary Figure 2. The expression of RhoGDIA at 10 μM of nicotine for different time.

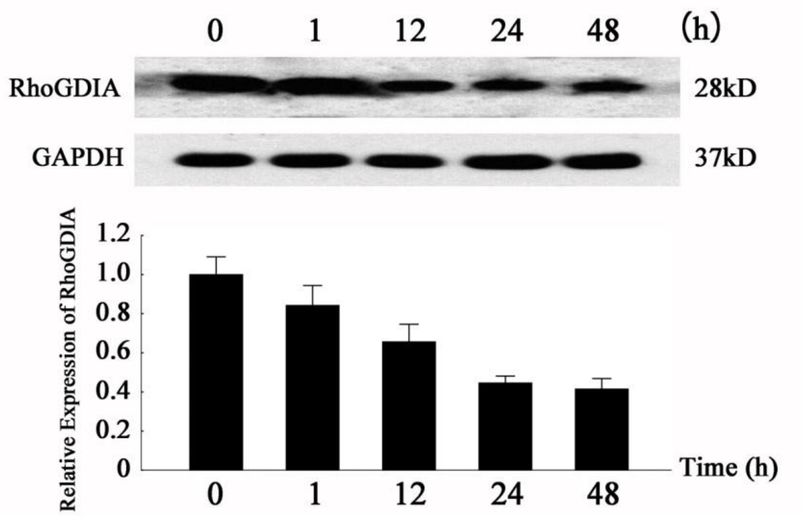

VSMCs were incubated with nicotine at concentration of 10 μM for various amounts of time (0, 1, 12, 24, 48 h). The expression of RhoGDIA was detected by western blotting. The values are expressed as the mean ± SEM (n=5).

Supplementary Figure 3. The measurements for changes on vascular function after nicotine treatment.

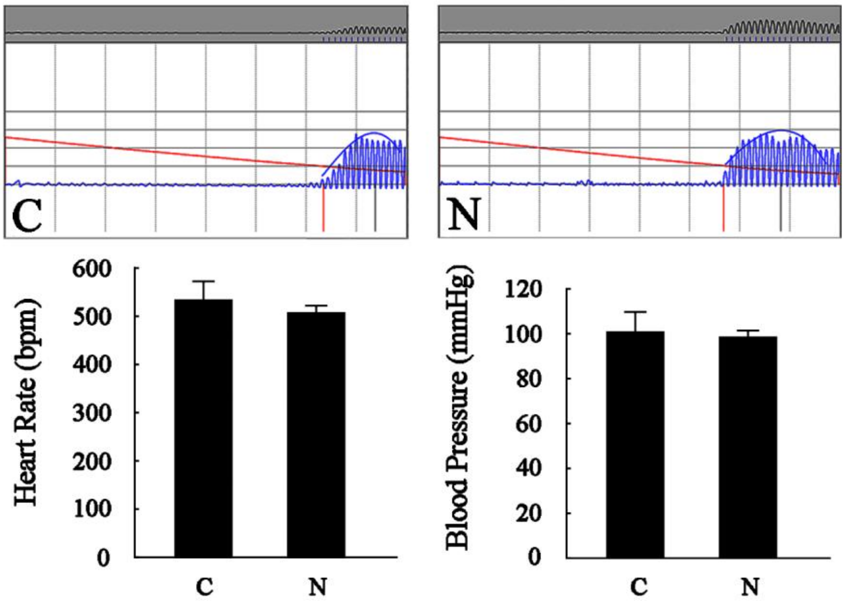

Blood pressure and heart rate were measured in the C57Bl/6J mouse by the tail-cuff method. Nicotine had only tiny influence on blood pressure and heart rate; however, there is no significant difference between two groups (Heart Rate: p=0.445; Blood Pressure: p=0.097). The values are expressed as the mean ± SEM.

Supplementary Figure 4. Immunohistochemical detection of RhoGDIA in the thoracic arteries of PBS- and nicotine-treated mice.

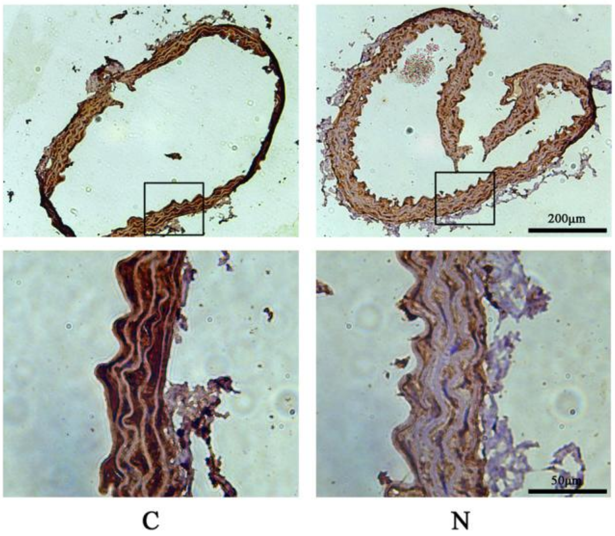

RhoGDIA was stained brown and DNA was stained blue. The bottom line shows the enlarged portion in the above rectangle.

Supplementary Figure 5. DNA methylation analyses of CpGs in the promoter region of *RhoGDIA* in control and nicotine-treated VSMCs.

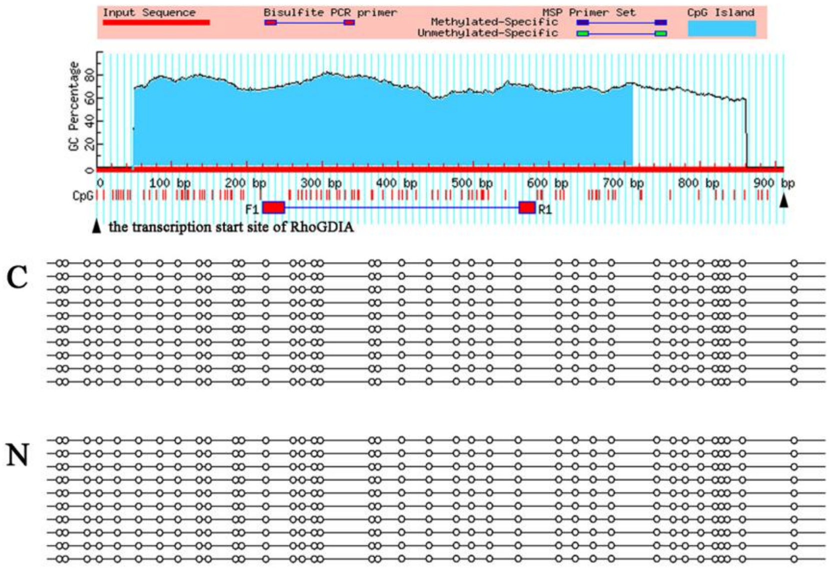

CpG islands are tinted blue. Each spot represents a methylation site (CpG) and the black spots indicate methylated Cs.

Supplementary Figure 6. Real-time PCR analysis of the expression level of Pri-miR-200b in VSMCs. The values are expressed as the mean ± SEM (n=5, **p < 0.01).

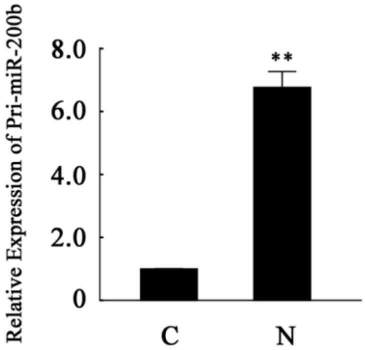

Supplementary Figure 7. Up-regulation of *miR-200b* was associated with DNA hypomethylation of the promoter region *in vivo*.

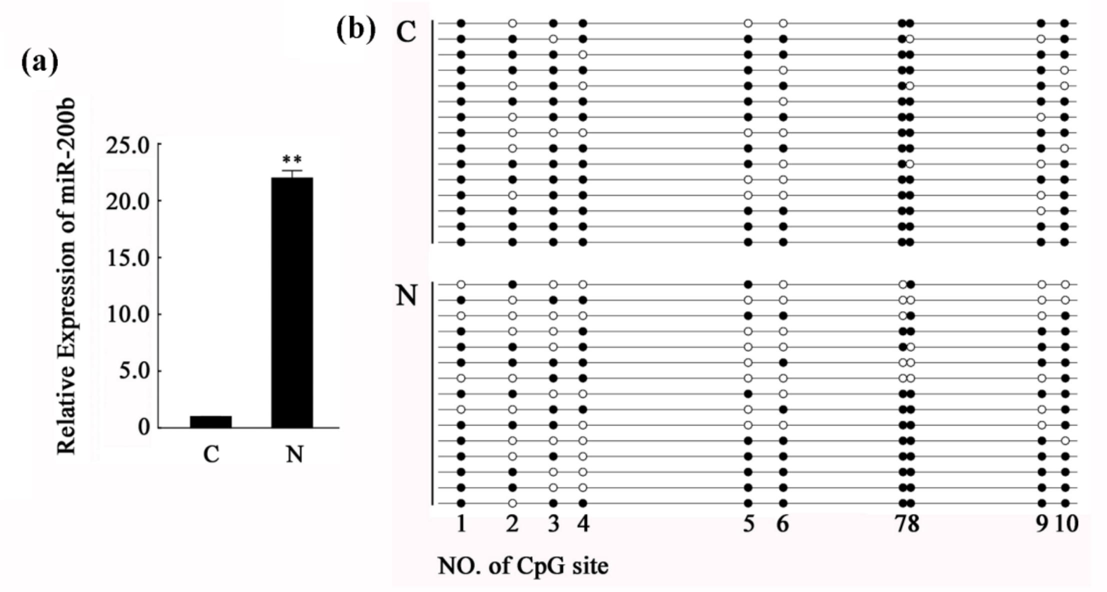

(a) Real-time PCR analysis of the expression level of miR-200b in the thoracic arteries of mice. (b) CpG methylation analysis of the thoracic arteries from the control and nicotine-treated mice. These results demonstrate that *miR-200b* undergoes epigenetic changes following nicotine treatment *in vivo*.

Supplementary Figure 8. The activity level and protein level of RhoA, RAC1 and CDC42 in VSMCs after transfection with either the miR-200b mimics or a scrambled control.

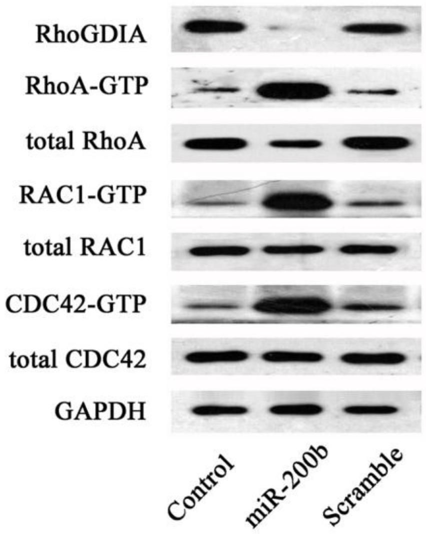

Supplementary Figure 9. The migration image of VSMCs treated with nicotine, miR-200b mimics (100 nmol/L) and miR-200b inhibitors (100 nmol/L).

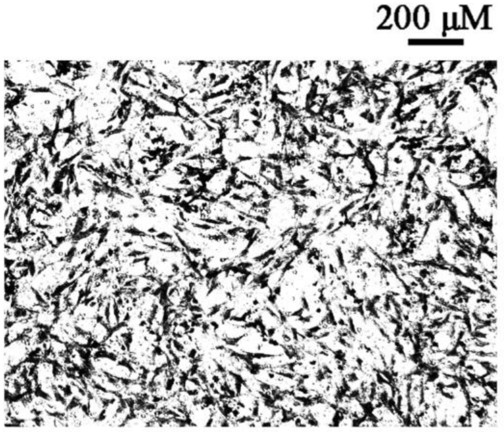

Supplementary Figure 10. Exploration of the mechanism underlying nicotine-induced RhoA change.

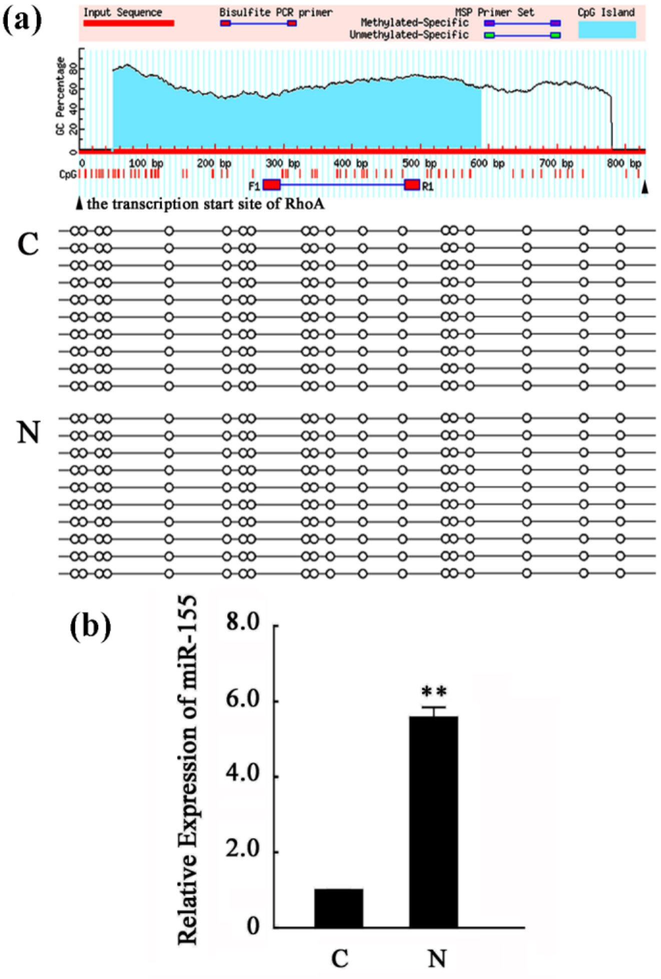

(a) DNA methylation analysis of CpGs in the *RhoA* promoter from control and nicotine-treated VSMCs. CpG islands are tinted blue. Each spot represents a methylation site (CpG) and the black spots indicate methylated Cs. (b) Real-time PCR analysis of the expression level of miR-155 in VSMCs. The values are expressed as the mean $\pm$ SEM (n=5, $^{**}p < 0.01$)
